# Prolonged Grief Disorder in a Diverse College Student Sample

**DOI:** 10.3389/fpsyg.2020.604573

**Published:** 2021-01-11

**Authors:** Kim Glickman

**Affiliations:** Department of Social Work, York College/City University of New York, NY, United States

**Keywords:** prolonged grief disorder, grief support, attachment, bereaved, students of color

## Abstract

**Objective**: The purpose of this study was to explore the rate of prolonged grief disorder (PGD) and associated factors in a large sample of diverse college students. Sources of grief support and perceived helpfulness of support were also examined.

**Method:** An online survey was administered to bereaved students at three colleges at the City University of New York. PGD measured by the Inventory of Complicated Grief was the primary outcome. Chi-squared and *t*-tests were used to assess the association between PGD and associated factors.

**Results**: A total of *n* = 899 participants completed the Inventory of Complicated Grief (ICG) based on a significant death loss = >12 months. An estimated 13.4% (*n* = 120/899) met criteria for PGD. The rate of PGD was associated with race, history of anxiety or depression, trauma other than the death, insecure attachment style, kinship to the deceased, closeness to the deceased, cause of death, and sudden/unexpected death. The majority of students sought grief support from a friend or family member.

**Conclusion**: The rate of PGD in this sample of college students is similar to that of adults and most prevalent for students of color. Identification of those most at risk is critical to referring these students to effective treatments.

## Introduction

The death of a loved one is one of the most difficult life experiences and often triggers a grief reaction with intense and painful emotions. While acute grief usually dissipates over time and becomes integrated, a sizeable minority of people has ongoing difficulty after the loss. Severe and prolonged grief symptomatology is designated as Persistent Complex Bereavement Disorder (PCBD) in the DSM-5 (American Psychiatric Association, 2013). Proposed revisions to modify criteria for “Prolonged Grief Disorder (PGD)” will soon recognize it as a formal DSM diagnosis within section Materials and Methods. The ICD-11 (World Health Organization, 2019) characterizes PGD as persistent symptoms of acute grief lasting beyond 6 months that include yearning for or preoccupation with thoughts of the deceased and intense emotional pain (e.g., sadness, guilt, anger, difficulty accepting the death, feeling one has lost a part of one’s self, an inability to experience positive mood, and emotional numbness). These symptoms cause significant impairment in work and social functioning and a withdrawal from previously enjoyed activities. Prevalence studies have shown that roughly 10% of adults bereaved by natural causes develop PGD (Lundorff et al., 2017) with higher rates for bereavement due to traumatic/violent death or disasters (Kristensen et al., 2012).

One third of traditional age college students (average age 18–23) in the United States have lost at least one family member or friend in the past year (Balk et al., 2010) and nearly half have lost a loved one in the past 2 years (Varga et al., 2015). Due to the Covid19 pandemic, where (at the time of this writing) over 300,000 lives have been lost in the United States, the number of bereaved is likely higher, and disproportionally impacts students of color. For all college students, bereavement can present unique challenges due to the pressures of juggling school, work, and family responsibilities, and for some, being separated from familiar support systems. Many bereaved students of color face additional challenges such as limited access to resources including access to quality and affordable healthcare.

Consequences of PGD for college students include lack of concentration and focus, lack of motivation, sleep difficulties, isolation from family and friends, suicidal ideation, poor grades, and possible dropout or dismissal, in addition to mental and physical health problems common to adults with PGD such as anxiety, depression, and chronic illness (Cox et al., 2015). PGD can persist for years without targeted intervention.

Limited research has been conducted on the prevalence of PGD in college students and these studies have significant limitations including small sample sizes, overly restrictive inclusion criteria (limiting bereavement to the past 2 years) and a lack of diverse samples.

Prevalence rates between studies are difficult to compare due to the use of different instruments and different criteria for measuring PGD. However, the rates of PGD in college students reported in prior studies are significantly lower than rates found in adults. Balk et al. (2010) found that 4.3% (*n* = 2/46) of predominantly white college students could be classified as having PGD using the 13-item Inventory of Traumatic Grief (ITG; Prigerson et al., 2008). Varga et al. (2015) found that 5.52% (*n* = 10/181) of bereaved college students from the United Arab Emirates and the Southeast United States met criteria for PGD utilizing the Prolonged Grief Questionnaire (PG-13; Prigerson et al., 2009). Varga (2016) found that only 0.5% (*n* = 6/1132) of graduate students in the Southeast Unites States met criteria for PGD utilizing the PG-13. All three of these studies defined bereavement as experiencing a death-loss within the past 2 years.

Lifetime death-losses were included in one study by Al-Gamal et al. (2019) of bereaved undergraduate students from Saudi Arabia. Using a cut-off score of 32 on the PG-13, with bereavement 6 months or longer, (*n* = 27/226) 12% met criteria for PGD. The highest rate of PGD was found by Williams et al. (2019) who examined bereavement-related mental health problems in undergraduates from a public Midwestern university with a history of sudden, unexpected death-loss. Using a cut-off score of 26 on the PG-13, (*n* = 46/326) 14% met criteria for PGD, however, time since loss was not specified.

The vast majority of studies on bereavement in American college students have used samples that were predominantly white. Only a handful of studies used more diverse samples and none of them reported the prevalence of PGD. However, among 1,581 bereaved students (40% Black) enrolled at a large Southern University, Laurie and Neimeyer (2008) found that Black students had higher levels of complicated grief symptoms (not PGD as a diagnosis) than whites and experienced more bereavement by homicide and greater grief for the loss of extended kin. Studies on bereavement in Black adults have shown that Blacks have higher rates of PGD than whites (21 vs. 12%; Goldsmith et al., 2008), more experiences of unexpected loss and high reliance on religious coping (Schoulte, 2011).

In addition to race (Goldsmith et al., 2008), prior research has identified a range of risk factors for PGD in adults such as sudden loss (Kristensen et al., 2012), violent/traumatic death (Djelantik et al., 2017), kinship with the deceased (loss of child or spouse; Newson et al., 2011), insecure attachment style (Jerga et al., 2011), history of mood or anxiety disorders (Melhem et al., 2004; Vanderwerker et al., 2006), prior exposure to trauma (Nickerson et al., 2014), age (Lundorff et al., 2017), and gender (Kersting, 2011).

Factors associated with PGD among college students, particularly students of color have been underexamined. Al-Gamal et al. (2019) found in Saudi Arabian bereaved students that PGD was associated with depression, lack of social support, and being female. In predominantly white American college student samples, higher grief symptoms (not PGD as a diagnosis) have been associated with insomnia, traumatic death, closeness to the deceased (Hardison et al., 2005), avoidant emotional coping (Cousins et al., 2017), and experiential avoidance (Murrell et al., 2018).

An examination of the rate of PGD in a more diverse college student sample might yield important information about the grief experiences of this population. Based on the findings reported by Goldsmith et al. (2008) and Laurie and Neimeyer (2008), we could expect to find a higher rate of PGD in this sample. Prior research points to several factors that might contribute to greater grief complications among students of color including higher rates of unexpected/sudden loss, violent death, closer attachments with extended family members (e.g., grandparents, cousins, aunts, and uncles), and prior traumatic events (Schoulte, 2011).

Researchers in the field of complicated/prolonged grief assert that problematic thoughts, such as ruminations about why or how the person died or behaviors such as avoidance of reminders of the loss can interfere with the normal healing process, resulting in a prolonged and intense state of acute grief (Shear, 2012). Certain risk factors (as mentioned above) can increase the likelihood that these problematic thoughts and behaviors will gain a foothold. College students may experience risk factors similar to adults; however, research on this topic is limited.

The purpose of this exploratory study is 3-fold: (1) to identify the rate of PGD in a large sample of diverse college students, (2) to examine the relationship between PGD and potential risk factors, and (3) to identify sources of grief support used by these students and perceptions of the helpfulness of these supports.

## Materials and Methods

### Participants

The sample comprised 974 students attending three colleges at a public university in a large northeastern metropolitan area during the 2019–2020 academic year. Internet survey data were collected over two semesters (at a single time point for each participant) through the college research pool for course credit. Participants had to be 18 years or older and have experienced the death of a family member or close friend at any point in their life. Students indicated their consent to participate after reading a description of the study on-line. Participants were provided with a list of grief resources at the end of the survey. The university’s institutional review board approved the study.

### Measures

**Socio-demographic characteristics** were measured by a questionnaire that included age, gender identity, race/ethnicity, religiosity/spirituality, academic status, and country of origin.

**Loss summary questionnaire** (Shear, 2009) was adapted to collect information about the number of deaths experienced and characteristics of the most significant death. For the most significant death, respondents were asked to provide the age of deceased, time since death, cause of death (illness, accident, homicide, and suicide), whether it was sudden/expected, and level of closeness of the relationship (four point scale from “not very close” to “extremely close”).

**History of depression, anxiety, and other traumatic events** were assessed with the following single-item questions: “Have you ever been diagnosed with depression? Have you ever been diagnosed with anxiety? Have you ever experienced a traumatic event, excluding the death of your loved one? Examples of traumatic events include but are not limited to physical or sexual assault, natural disasters, serious accident, life threatening illness, being in combat while in military service, physical or sexual abuse, or witnessing an assault or death of another person.”

**Inventory of Complicated Grief (ICG)** is a 19-item self-report measure of clinically impairing grief symptoms (Prigerson et al., 1995). The ICG has been used in various studies of CG to determine severity of grief symptoms and has good internal validity and reliability (alpha = 0.94) and 6-month test-retest reliability (*r* = 0.80). Cronbach’s alpha in the present sample = 0.92. A diagnosis of PGD was based on three criteria: (1) bereavement for 12 months or longer, (2) ICG total score = >30, and (3) a score = >10 on the Work and Social Adjustment Scale (WSAS). While the authors of the ICG originally recommended a cut-off score of 25 to diagnose of PGD, further analysis of the instrument (Carmassi et al., 2014) recommended a more conservative value of 30 to increase sensitivity and specificity. This threshold has been used in various clinical trials of complicated grief treatment (Shear et al., 2016) and research studies on PGD (Treml et al., 2020).

**WSAS** is a modification of a scale developed by Hafner and Marks (1976), which measures grief-related functional impairment. It is a five-item scale with ratings of 0 (not at all) to 8 (severe interference) on the extent to which grief interferes in five areas of daily functioning: work, home management, private leisure, social leisure, and family relationships. It has good internal validity and reliability (alpha = 0.94) and test-retest reliability (r = 0.73). Cronbach’s alpha in the present sample = 0.93. Scores of 10 or higher on the WSAS are associated with “significant functional impairment” (Mundt et al., 2002).

**Social Support Survey Instrument (SSS)** is a 19-item self-report scale that measures four dimensions of social support: emotional/informational (e.g., someone to confide in or talk to about yourself or your problems), tangible support (e.g., someone to help you if you were confined to bed); affectionate support (someone to love and make you feel wanted), and positive social interaction (e.g., someone to do something enjoyable with). Each item is scored on a five-point scale (0 = none of the time, 1 = a little of the time, 2 = some of the time, 3 = most of the time, and 4 = all the time). The scale has good internal consistency reliability for all subscales as well as the overall support index with alphas = 0.91–0.97 (Sherbourne and Stewart, 1991). Cronbach’s alpha in the present sample = 0.97 for the overall score and 0.95–0.97 for the subscales. A binary variable for social support was derived so that participants with a composite score of >38 were considered to have “good social support” while those with a score = <38 had “poor social support.” This threshold was based on having an average score of 2 or less on each question in the scale.

**Experiences in Close Relationships Scale (ECR-Short Version)** is a 12-item self-report scale that measures two dimensions of attachment style: attachment anxiety (e.g., I worry that others will not care about me as much as I care about them) and attachment avoidance (e.g., I am nervous when another person gets too close to me). Each item is rated on a 7-point scale from 1 (strongly disagree) to 7 (strongly agree). The measure has adequate internal consistency {alpha = 0.78 (anxiety) and 0.84 (avoidance) and good test-retest reliability [*r* = 0.82 (anxiety) and *r* = 0.89 (avoidance)]; Wei et al., 2007}. Cronbach’s alpha in the present sample = 0.68 for anxiety and 0.75 for avoidance.

**Grief Support** was assessed with the following question: “Think about the people to whom you have turned for support regarding your grief. Please indicate whether you sought help from the following and if so, their level of helpfulness (not at all helpful, a little helpful, and very helpful): religious leader, licensed mental health professional at your college counseling center, licensed mental health professional in the community, doctor/general practitioner, family member, friend, or other.” A qualitative question was also asked about “any other ways you have tried to cope with your grief” and “how helpful each activity was in coping with your grief.”

### Statistical Analysis

STATA version 16.1 was used for all statistical analyses. PGD was the primary outcome. Chi-square tests (categorical) and *t*-tests (continuous) were used to assess the association between PGD and demographic and psychosocial variables. Descriptive statistics were stratified by PGD diagnosis. Statistical significance was set at *p* < =0.05 for all analyses.

## Results

### Participant Characteristics

Demographic and psychosocial characteristics stratified by PGD are shown in Table 1. Sixty-nine percent of participants were female with a mean age of 22 years (range 18–60). The sample was 31% Asian, 30% Hispanic, 24% Black (non-Hispanic), and 13% White (non-Hispanic). Thirty-eight percent were born outside the United States, 23% had a history of depression or anxiety, and 46% experienced a traumatic event other than the death.

**Table 1:** Demographic/psychosocial characteristics by prolonged grief disorder (PGD).

	Total *N* = 899	No PGD (*n* = 779)	PGD (*n* = 120)	*p*
**Continuous variable**				
Mean age (SD)	22.0 (6.2)	22.0 (6.3)	21.9 (5.5)	0.93
Mean anxious attachment (SD)	21.6 (6.4)	21.2 (6.4)	24.9 (5.1)	<0.001
Mean avoidant attachment (SD)	24.4 (7.1)	23.9 (7.2)	27.5 (5.2)	<0.001
**Categorical variable**	**Total *n***	***n* (%)**	***n* (%)**	
Race/ethnicity				0.04
White, non-Hispanic	114	104 (91.2)	10 (8.8)	
Black, non-Hispanic	215	179 (83.3)	36 (16.7)	
Hispanic	267	239 (89.5)	28 (10.5)	
Asian	272	229 (84.2)	43 (15.8)	
Native American/Hawaiian	22	21 (95.5)	1 (4.6)	
Gender				0.15
Male	270	243 (90.0)	27 (10.0)	
Female	621	529 (85.2)	92 (14.8)	
College				0.24
Baruch College	306	272 (88.9)	34 (11.1)	
City College	189	158 (83.6)	31 (16.4)	
York College	404	349 (86.4)	55 (13.6)	
Religious/spiritual				0.84
Not at all	160	142 (88.8)	18 (11.3)	
A little	247	212 (85.8)	35 (14.2)	
Moderately	394	341 (86.6)	53 (13.5)	
Extremely	98	84 (85.7)	14 (14.3)	
Academic year				0.80
Freshman	318	279 (87.8)	39 (12.3)	
Sophomore	179	154 (86.0)	25 (14.0)	
Junior	262	223 (85.1)	39 (14.9)	
Senior	128	111 (86.7)	17 (13.3)	
Born in United States				0.74
No	340	293 (86.2)	47 (13.8)	
Yes	559	486 (86.9)	73 (13.1)	
History of anxiety or depression				<0.001
No history	691	616 (89.2)	75 (10.9)	
Positive history	204	160 (78.4)	44 (21.6)	
History of other trauma				0.01
No	483	431 (89.2)	52 (10.8)	
Yes	414	346 (83.6)	68 (16.4)	
Social support				0.19
Good support	791	695 (87.9)	96 (12.1)	
Poor support	68	56 (82.4)	12 (17.7)	

Not all categories add up to 899 due to missing data.

### Loss Characteristics

Loss characteristics were stratified by PGD and are shown in Table 2. More than one loss was reported by 76% of the sample. Most significant loss included a grandparent (53%), aunt/uncle (17.5%), friend (11%), parent (9%), sibling (2%), spouse/significant other (0.5%), and other (e.g., cousin, niece/nephew, and godfather/godmother; 7%). Average time since death was 6 years (range 0–38). Cause of death was illness (80%), accident (13%), homicide (4%), and suicide (3%). Sudden/unexpected death was reported by 69%. Thirty-six percent reported being “not all or somewhat close” to the deceased, 38% “very close,” and 26% “extremely close.” Mean ICG score was 19.23, SD = 13.12 and mean WSAS score was 5.72, SD = 7.76. The overall rate of PGD was 13.4% (*n* = 120/899).

**Table 2:** Loss variables by PGD.

	Total *N* (*n* = 899)	No PGD (*n* = 779)	PGD (*n* = 120)	*p*
**Continuous variable**				
Mean years since death	6.1 (5.1)	6.1 (4.9)	6.1 (6.1)	0.52
Mean Inventory of Complicated Grief (ICG)	19.2 (13.1)	16.0 (8.3)	40.3 (8.3)	<0.001
Mean Work and Social Adjustment Scale (WSAS)	5.7 (7.8)	3.8 (6.0)	18.5 (5.9)	<0.001
**Categorical variable**	**Total *n***	***n* (%)**	***n* (%)**	
Number of losses				
One	211	188 (89.1)	23 (10.9)	0.45
Two	281	240 (85.4)	41 (14.6)	
Three or more	401	345 (86.0)	56 (14.0)	
Most significant loss				<0.001
Parent	84	59 (70.2)	25 (29.8)	
Grandparent	470	424 (90.2)	46 (9.8)	
Significant other	5	2 (40.0)	3 (60.0)	
Aunt or uncle	157	141 (89.8)	16 (10.2)	
Sibling	17	13 (76.5)	4 (23.5)	
Friend	101	83 (82.2)	18 (17.8)	
Other^*^	65	57 (87.7)	8 (12.3)	
Closeness to deceased				<0.001
Not at all/somewhat	319	300 (94.0)	19 (6.0)	
Close	343	294 (85.7)	49 (14.3)	
Very close	237	185 (78.1)	52 (21.9)	
Extremely close				
Cause of death				<0.001
Illness	712	634 (89.0)	78 (11.0)	
Accident	111	87 (79.4)	24 (21.6)	
Homicide	37	30 (81.1)	7 (18.9)	
Suicide	30	21 (70.0)	9 (30.0)	
Sudden/unexpected death				0.01
No	280	255 (91.1)	25 (8.9)	
Yes	619	524 (84.7)	95 (15.4)	

Not all categories add up to 899 due to missing data.

* Other significant loss includes cousin, niece, nephew, godparent, and mother-in-law.

### Association Between Personal Characteristics and PGD

Having PGD was associated with race/ethnicity, *X*^2^ (5, N = 895) = 12.02, *p* = 0.04, whereby the rate of PGD among Black students (16.7%) was twice that of white students (8.8%). The rates of PGD among Asians (15.8%) and Hispanics (10.5%) were also higher than that of whites.

Higher rates of PGD were also associated with the history of depression or anxiety, *X*^2^ (1, N = 895) = 15.7, *p* < 0.001, history of trauma other than the death, *X*^2^ (1, N = 897) = 6.2, *p* = 0.01, and attachment style: anxious, *t*(882) = −5.97 *p* < 0.001 and avoidant, *t*(882) = −5.03, *p* < 0.001.

### Association Between Relationship With the Deceased and PGD

PGD was associated with kinship, *X*^2^ (6, N = 899) = 38.8, *p* < 0.001, whereby rates were higher for those who had lost a significant other (60.0%), parent (29.8%), or sibling (23.5%) than those who had lost a friend (17.8%), aunt/uncle (10.2%), or grandparent (9.8%). PGD was also associated with closeness to the deceased, *X*^2^ (2, N = 899) = 30.5, *p* < 0.001.

### Association Between Circumstances of the Death and PGD

The rate of PGD was higher for those who were bereaved due to suicide (30.0%) than for those who were bereaved due to accident (21.6%), homicide (18.9%), or illness (11.0%), *X*^2^ (3, N = 890) = 18.3, *p* < 0.001. PGD was associated with sudden/unexpected death, *X*^2^ (1, N = 933) = 9.0, *p* = 0.003.

There were no statistically significant associations between PGD and age, gender, religiosity/spirituality, college attended, academic year, being born in the United States, social support, number of losses, or years since the death.

### Grief Support

Sources of grief support and perceived helpfulness are shown in Table 3. The majority of participants sought help from a friend (80%) or family member (76%). The least utilized support person was a professor (11%) and college mental health counselor (14%). The helper with the highest rating for “how helpful was the helper?” was religious leader (62%) although this source of support was only utilized by 16% of participants.

**Table 3:** Sources of grief support and perceived helpfulness.

Source of grief support	Utilized (%)	Very helpful (%)
Friend	80	59
Family member	76	55
Other helper^*^	54	7
Community mental health counselor	21	46
Doctor/GP	19	38
Religious leader	16	62
College mental health counselor	14	37
Professor	11	54

* Other helper includes boss, boyfriend/girlfriend, coach, co-worker, God, and pet.

Other sources of comfort/coping included exercise (sports, yoga, dance, and walking), meditation, visual arts, listening to and playing music, writing in a journal, writing to the deceased, watching TV/movies, reading, playing video games, looking at old pictures/videos/text messages of the deceased, using social media, housework/chores, spending time with family and friends, talking to the deceased, caring for plants/animals, God/religion, therapy, support groups, charity work (helping others), time outdoors, crying/yelling, staying busy, visiting the cemetery, and traveling. One student wrote,

I know that (my loved one) loved to write poems and songs; therefore, I sometimes write about her and life in general. Sometimes I sing and listen to the songs she taught me. I honor her memory by wearing her skirt on her birthday. When I was a child, I watched her sew the skirt and add beautiful embroideries by hand. I feel that wearing that skirt is special and it captures her essence and creativity, in many ways that live in me. All this has helped me to accept her death and not think about the ways that I was absent. I think about the wonderful person that she was, and I look for ways that her memory lives on. Remembering her is my way of grieving.

## Discussion

To this author’s knowledge, this study is the first to report on the prevalence of PGD in a large sample of diverse American college students. The overall rate of PGD was 13.4%, higher than what has been reported in college samples, particularly when bereavement was limited to the past 2 years (0.05–5.5%). Balk et al. (2010) speculated that the relatively low rate of PGD found in their sample may have been due to under-reporting (e.g., seeing themselves as not needing help or being too impaired to participate in a research study). Alternatively, the authors speculated that resilience among college students may buffer them from developing PGD. Varga et al. (2015) posited that the low rate of PGD (5.52%) found in their sample may have been due to the absence of students who had failed or dropped out of school. The results in the present study suggest a couple of additional explanations; that the low rate of PGD found in these studies may have been due to limiting time since death to the last 24 months or that predominantly, white college students are less vulnerable to PGD than students of color.

In contrast with the aforementioned studies, the present study included lifetime death-losses, which more accurately captures the number of people with PGD. The average time since death in this sample was 6 years, suggesting that studies that have limited bereavement to the last 2 years have grossly underestimated the number of students suffering with the disorder.

The rate of PGD of 13.4% found in this study is consistent with what has been found for adults. A meta-analysis of 14 studies revealed a pooled prevalence of PGD of 9.8% in people bereaved by natural causes although a variety of measurement tools and criteria for PGD were used across studies (Lundorff et al., 2017). The results of the present study may suggest that young adults are no less vulnerable to PGD than older adults. Another possibility is that PGD is more prevalent among students of color than among white students. Further studies that include lifetime death-losses and diverse samples should be conducted to confirm both of these hypotheses.

The rate of PGD among Black students (16.7%) was higher than any other group and nearly twice the rate of white students (8.8%). This is consistent with findings reported by Goldsmith et al. (2008), where 21% of Black adults had PGD compared with 12% of white adults. A host of environmental factors probably contribute to racial/ethnic disparities in grief outcomes. The accumulation of daily stressors caused by microaggressions and systemic racism, and inadequate access to psychological and social services, quality healthcare and essential material support, can magnify stress following the loss of a loved one. If financial circumstances of the bereaved change as a consequence of the death (e.g., loss of income, housing, etc.), this can be an additional burden. Higher death rates in the Black community due to natural and unnatural causes (Schoulte, 2011) may also contribute to higher rates of PGD among students of color. Black students in this sample were more likely than white students to experience sudden loss, (OR = 1.89; 95% CI = 1.19–3.01; *p* = 0.007), bereavement by homicide (OR = 13.65; CI = 1.82–102.29; *p* = 0.011) and have a history of trauma other than the death (OR = 1.64; CI = 1.05–2.54; *p* = 0.029). Stress theorists note that exposure to chronic stress has long-term physiological effects, which negatively impact both physical and psychological health (Clark et al., 1999; Gouin et al., 2008). As a result of the chronic stress that many people of color in the United States experience (Price et al., 2010), the death of a loved one may overwhelm an already overtaxed coping system and increase vulnerability to grief complications.

Although history of depression or anxiety was not measured with validated instruments, the results presented here are consistent with prior research with both adolescents (Melhem et al., 2007) and adults (Vanderwerker et al., 2006; Nickerson et al., 2014). PGD is often accompanied by comorbid disorders such as depression, anxiety, and PTSD (Keyes et al., 2014), which may be risk factors for PGD or consequences of the death. However, a longitudinal study on parentally bereaved children and adolescents found that prolonged grief was predicted by a previous history of depression (Melhem et al., 2011), providing evidence that at least the former may be true.

Consistent with prior research, attachment anxiety and attachment avoidance were both associated with PGD (Fraley and Bonanno, 2004; Field and Filanosky, 2010; Meert et al., 2010; Jerga et al., 2011). Those who lost a parent, significant other or sibling had the highest rates of PGD. For these students, it is possible that attachment insecurity increased their vulnerability to grief complications. It is also possible that given their young age, the death of their loved one resulted in a more insecure attachment style rather than the reverse. The direction of this relationship requires further study using both trait and state measures of attachment and a longitudinal research design.

Whereas several studies with adults have shown that losing a spouse or a child increases the risk for prolonged grief (Kersting, 2011; Newson et al., 2011), the present study found that losing a parent was associated with a high rate of PGD (30%) compared with other losses. That nearly one-third of those who lost a parent developed PGD speaks to the enormity of this loss for young people. It is also consistent with theoretical frameworks for prolonged grief, particularly attachment theory, which emphasizes the severe disruption (both psychological and physiological) caused by the loss of an attachment figure, particularly parental loss for children (Bowlby, 1980). Also consistent with attachment theory is the finding that PGD was associated with closeness to the deceased. Most people who develop PGD describe having had a close and loving relationship with the person who died (Shear, 2012).

Circumstances of the death such as sudden/unexpected loss and loss due to suicide were also associated with PGD. In a literature review of bereavement and mental health after sudden and violent losses, Kristensen et al. (2012) point to the difficulty of “grasping the reality” that a loved one has died when the loss is unexpected. Sudden loss deprives the bereaved of an opportunity to say goodbye or carry out any last wishes. If circumstances of the death were traumatic (e.g., violent) or distressing (e.g., loved one was alone or in physical pain), the bereaved may suffer with persistent troubling thoughts about the loved one’s last moments. Suicide-loss presents unique challenges for loved ones who often experience feelings of rejection, stigma, shame, and self-blame. Rates of suicide among the suicide-bereaved are also higher than for non-suicide bereaved, making this a particularly vulnerable subgroup (Tal et al., 2017).

For grief support, participants sought help primarily from a friend (80%), family member (76%), and “other helper” (54%), which included God, a boss, boyfriend/girlfriend, coach, coworker, and a pet. The least utilized supports were doctor, mental health counselor, religious leader, and professor. That 80% of the sample sought help from friends and only 60% found this support helpful suggests that colleges could better address the needs of this population by educating students about grief and how to respond to classmates who are bereaved.

There are several limitations of this study. The sample was drawn from a college research pool available mainly to social science students who self-selected and may not be representative of the general population of college students. Data were collected at only one time point (after the loss), which makes it impossible to assess the directionality of the relationship between PGD and associated factors. History of depression/anxiety diagnosis and history of trauma were measured with single-item questions, which may limit the reliability of these variables. The use of self-report measures across the board may not be as reliable as clinician-reports due to distortion on the part of subjects. Finally, socio-economic status and financial consequences of the death were not included in the analysis, which could provide a more nuanced understanding of the rate of PGD in this sample. CUNY is a public university, accessible to students of all economic backgrounds. Forty percent of its students come from households that earn less than $20,000 per year and over 90% come from households considered to be low-income^1^ (CUNY Office of Institutional Research, 2016). Prior research has shown that lower income is associated with PGD in adults (Kersting, 2011) and college students (Al-Gamal et al., 2019). It would be important to tease out the effects of income/financial strain from race/ethnicity, which may be conflated in this study.

Despite these limitations, this is the first study to examine the rate of PGD in a large college student sample that includes a majority of students of color and takes into account lifetime death-losses. Results showed an overall rate of PGD that was higher than what has been reported for predominantly white college student samples. Results presented here also revealed a large disparity in rates of PGD between students of color and white students, providing further evidence of worse health outcomes for people of color in the United States.

Screening of high-risk students should be conducted on college campuses and targeted treatment for PGD should be made available for those who need it. Mental health counselors at college counseling centers could be trained in evidence-based approaches such as Complicated Grief Treatment (Shear et al., 2016), which can be offered in 16 sessions, suitable to a transient student population. Peer education on grief support would also contribute to better outcomes for bereaved college students who more frequently turn to peers than counselors when feeling distressed.

Additionally, several important demographic and psychosocial factors should be viewed as potential risk factors when screening for PGD including race/ethnicity, history of depression/anxiety and trauma, insecure attachment style, kinship, closeness to the deceased, sudden/unexpected death, and death due to suicide. College counselors and peer educators should be alert for these factors in order to identify and refer bereaved students who may be most at risk for PGD.

## Data Availability Statement

The raw data supporting the conclusions of this article will be made available by the author, without undue reservation.

## Ethics Statement

The studies involving human participants were reviewed and approved by the University Integrated Institutional Review Board of The City University of New York. The participants provided their written informed consent to participate in this study.

## Author Contributions

KG conducted all aspects of the project including research design, data collection, data analysis, preparation of the manuscript, and revisions.

### Conflict of Interest

The author declares that the research was conducted in the absence of any commercial or financial relationships that could be construed as a potential conflict of interest.

## Footnotes

^1^Low-income is defined as households earning less than 80% of the area medium income (AMI). The AMI of the New York City region (established by the Department of Housing and Urban Development) is $113,700 for a family of four (New York City Housing Development Corporation, 2020).
